# Intravesical alprostadil as a promising agent in BK virus-associated hemorrhagic cystitis: A report of a refractory case

**DOI:** 10.5339/qmj.2021.51

**Published:** 2021-10-07

**Authors:** Shahin Shamsian, Ali Saffaei, Fatemeh Malek, Zahra Khafafpour, Abtin Latifi, Mahdieh Karamat, Bahador Mirrahimi

**Affiliations:** ^1^Pediatric Congenital Hematologic Disorders Research Center, Mofid Children's Hospital, Shahid Beheshti University of Medical Sciences, Tehran, Iran E-mail: bahador.p.d@gmail.com; ^2^Student Research Committee, Department of Clinical Pharmacy, School of Pharmacy, Shahid Beheshti University of Medical Sciences, Tehran, Iran; ^3^Department of Clinical Pharmacy, School of Pharmacy, Shahid Beheshti University of Medical Sciences, Tehran, Iran

**Keywords:** allogeneic stem cell transplant, alprostadil, BK virus, hemorrhagic cystitis

## Abstract

Allogeneic stem cell transplant recipients are at risk of BK virus-associated hemorrhagic cystitis. This condition causes a significant morbidity and worsens clinical outcomes. The standard cares for BK virus-associated hemorrhagic cystitis are saline irrigation and forced diuresis. Notably, several beneficial roles are proposed for antiviral and anti-inflammatory agents against BK virus-associated hemorrhagic cystitis. However, cases who are at risk of cystectomy remain refractory. Herein, we present a 13-year-old boy with severe hematuria by passing two months from his allogeneic stem cell transplantation. The laboratory work up showed high BK viremia >1.1 ×  10^8^ copies/ml in this case's urine sample. The patient was treated with antiviral agents in combination with supportive care. Moreover, intravesical alum was administered, but no clinical benefits were achieved. Finally, intravesical alprostadil was prepared under the supervision of a pediatric clinical pharmacist. In this regard, an alprostadil solution was prepared by constitution of 250 μg alprostadil in 50 mL saline. After administrating the first dose of intravesical alprostadil, an acceptable clinical response was observed, and hematuria stopped. Of note, alprostadil induces platelet aggregation and vasoconstriction. Thus, bleeding can be controlled after the administration of intravesical alprostadil. This strategy may be associated with several side effects including bladder spasm. This study is the first report describing the special role of intravesical alprostadil in refractory cases of BK virus-associated hemorrhagic cystitis. In such refractory cases, clinicians can use intravesical alprostadil rather than invasive therapies in the treatment of BK virus-hemorrhagic cystitis.

## Introduction

BK virus is mostly observed in childhood and remains in a latent state for whole life. BK virus reactivation occurs during the immunodeficiency phase, in which immunosuppressive agents increase the risk of BK virus replication. Allogeneic stem cell transplant (ASCT) recipients are at risk of BK virus-associated hemorrhagic cystitis. ^1^ The suggested pathogenesis of BK virus associated-hemorrhagic cystitis involves three distinct phases: 1) chemotherapy/irradiation damages the uroepithelium and decreases BK virus-specific cellular immunity; 2) thereafter, BK virus replicates during the immunosuppressive phase; finally, 3) the immune system attacks and cause additional damages to the uroepithelium after hematopoietic reconstitution. ^2^

A poor clinical outcome is expected in these patients. Intensive hydration and forced diuresis are effective strategies proposed for this situation. ^3^ The treatment of BK virus-associated hemorrhagic cystitis is still controversial. Antiviral and anti-inflammatory agents have certain benefits in this regard. ^4^ Notable attention has been directed toward the use of cidofovir in BK virus cases. Savona et al. showed that 84% of patients affected by this virus clinically responded to cidofovir; however, 47% of them had a decreased viral load in the urine. ^5^ Moreover, Tang et al. demonstrated that surgical treatments, including embolization and mucous electrocoagulation, are safe and effective methods for severe refractory hemorrhagic cystitis. ^6^ Cidofovir, intravenous immunoglobulins, and fluoroquinolones are among the systemic agents used in the treatment of BK virus-associated hemorrhagic cystitis. However, they are associated with several systemic side effects. Therefore, the treatment of refractory cases with the lowest number of side effects remains a clinical challenge.^1^ Correspondingly, surgical approaches (cystoscopic clot evacuation, embolization, and cystectomy) and medical treatments are among the current available options for the treatment of refractory cases. Furthermore, medical treatments, including intravesical agents such as E-aminocaproic acid, alum, silver nitrate, and formalin, are associated with specific significant side effects. Prostaglandins, such as alprostadil, are another option to control hemorrhagic cystitis. Accordingly, alprostadil acts as a vasoconstrictor agent, and its clinical applicability has been demonstrated in cyclophosphamide-induced hemorrhagic cystitis.^7^ Moreover, this drug can be administered through intravesical rout and has no significant side effects in comparison with systematic agents.

In the current study, we report the successful treatment of BK virus-associated hemorrhagic cystitis in an ASCT recipient with intravesical alprostadil.

## Case Presentation

**Figure 1.**
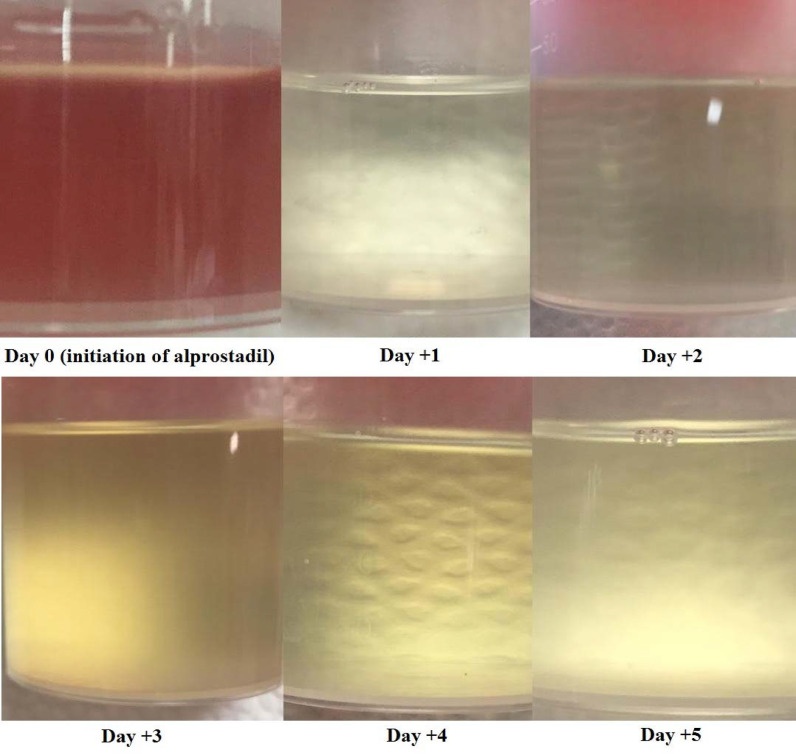
Bloody appearance of the urine obtained from a 13-year-old boy. The patient was diagnosed with BK virus-associated hemorrhagic cystitis following allogeneic stem cell transplantation. All supportive and recommended treatments failed to resolve the hemorrhage. Intravesical alprostadil was finally initiated. Acceptable clinical responses were observed, and the hematuria stopped. The bloody appearances of urine were observed in subsequent days.

A 13-year-old boy diagnosed with acute myeloid leukemia-M4 was admitted to the bone marrow transplantation ward for ASTC. He was at the complete remission stage at the time of admission. In addition, the patient had no medical history of other diseases. For this case, busulfan, cyclophosphamide, and melphalan were used as conditioning regimen. In addition, neutrophil and platelet engraftment occurred on day +10, and the patient was discharged with a stable clinical condition on day +15. Cyclosporine at dose of 50 mg p.o. was administered twice a day for graft versus host disease prophylaxis. All antimicrobial prophylaxes were also considered. Thereafter, on day +52, the patient had complaints of hematuria and was thus admitted to hospital again. In this regard, intensive fluid therapy and forced diuresis were considered along with the bladder irrigated with the continuous infusion of saline. The initial laboratory workup showed anemia, 10 g/dL hemoglobin, and 22000/mL platelets. High BK viremia >1.1 ×  10^8^ copies/mL in urine was also reported, but a negative result was observed in the blood sample. According to the clinical and paraclinical findings, the patient was diagnosed with BK virus-associated hemorrhagic cystitis. Cidofovir was unavailable in the pharmaceutical market, and valganciclovir was initiated at dose of 750 mg p.o. per day. In addition, leflunomide and intravenous immunoglobulin G were considered. Notably, the dose of cyclosporine, which is used prophylactic immunosuppression, decreased. Despite the supportive care and antiviral administrations, hemorrhagic cystitis continued for the next seven days. The hemoglobin levels decreased to 8.5 g/dL. Hence, intravesical alum (aluminum ammonium sulfate or aluminum potassium sulfate), which was prepared under the supervision of a clinical pharmacist, was initiated, and the patient was started on alum irrigation at 50 mL per day. Subsequently, the supportive care in combination with alum was continued for 10 days, but no clinical improvement was observed during this time. The hematuria was persistent, and the hemoglobin level decreased to 5 g/dL despite the two units of blood transfusion. Thus, the patient was transferred to the intensive care unit. All the above-mentioned treatments failed to improve the clinical situation of the patient, and during these treatments, the hemoglobin level was below than 8 g/dl. The patient also needed several blood transfusions during these days. To conclude, the patient was unresponsive to supportive cares, leflunomide, intravenous immunoglobulin G, valganciclovir, and alum. Therefore, a cystectomy operation was decided. However, prior to the operation, a last resort intravesical alprostadil was administered instead of an invasive cystectomy. Alprostadil solution was prepared by the constitution of 250 μg alprostadil in 50 mL saline. After administrating the first dose of intravesical alprostadil, an acceptable clinical responses was observed, and hematuria stopped. Given that no blood clots were reported by the patient during urination, the administration of alprostadil continued for the next several days. Figure 1 depicts the successful treatment over 5 days. The hemoglobin levels increased to 12 g/dL. Hence, no intervention of blood transfusion was deemed necessary. The patient was discharged on day 75 under acceptable clinical conditions.

## Discussion

BK virus can cause hemorrhagic cystitis in ASCT patients, and this condition is associated with a high morbidity rate and consequently the increased length of stay in hospital. In the case presented in this study, hemorrhagic cystitis developed after 2 months of transplantation. The standard care methods for the management of BK virus-associated hemorrhagic cystitis are supportive irrigation and blood product replacement. Certain medications, such as cidofovir, leflunomide, valganciclovir, and intravenous immunoglobulin G, present antiviral activities against BK virus. However, specific cases affected by this virus may be refractory to these treatments. Cystectomy, a challenging and morbid procedure, is a salvage strategy in several cases. This condition is associated with a high morbidity in children, and several complications are expected.^8,9^ Intravesical prostaglandin causes platelet aggregation and vasoconstriction to reduce bleeding.^10^ Moreover, it can reduce the inflammatory response by reducing histamine release and the effectiveness of cell-mediated inflammation.^11^ Alprostadil, which is a synthetic form of prostaglandin E1, was used in previous studies with a lack of confirmatory results.^12^ Previous studies suggested that the measurement of specific biomarkers can be helpful in patients who receive alprostadil. This process can help physicians to assess the efficacy of alprostadil. High-sensitivity CRP and homocysteine level are inflammatory mediators in this regard. Patients receiving alprostadil are monitored by measurements of their platelet count and careful analysis of their peripheral blood smear to assess the efficacy and safety of alprostadil.^13,14^ Although, our case did not respond to supportive treatment, such as saline irrigation, alum, and diuresis. The positive control of hematuria was observed with intravesical alprostadil. Alprostadil has multi mechanisms that support its rational usage. Alprostadil acts as an agonist to prostaglandin receptor (EP2). This process activates adenylate cyclase and causes the accumulation of 3′5′-cyclic adenosine monophosphate. This compound is responsible for the pharmacologic effects of alprostadil, including smooth muscle relaxation, bronchodilation, and inhibition of platelet aggregation.^15^ The patients mostly manifest no significant side effects with the use of intravesical alprostadil. However, previous studies reported bladder spasm in several cases in.^16^ In light of our findings, we can confidently conclude the beneficial effects of intravesical alprostadil in refractory cases. This novel treatment is highly recommended in children as a plausible alternative to cystectomy operation.
